# Neuroprotective and Antiapoptotic Activity of Lineage-Negative Bone Marrow Cells after Intravitreal Injection in a Mouse Model of Acute Retinal Injury

**DOI:** 10.1155/2015/620364

**Published:** 2015-02-24

**Authors:** Anna Machalińska, Dorota Rogińska, Ewa Pius-Sadowska, Miłosz P. Kawa, Edyta Paczkowska, Michał Rudnicki, Renata Lejkowska, Bartłomiej Baumert, Barbara Wiszniewska, Bogusław Machaliński

**Affiliations:** ^1^Department of Histology and Embryology, Pomeranian Medical University, Al. Powstancow Wlkp. 72, 70-111 Szczecin, Poland; ^2^Department of Ophthalmology, Pomeranian Medical University, Al. Powstancow Wlkp. 72, 70-111 Szczecin, Poland; ^3^Department of General Pathology, Pomeranian Medical University, Al. Powstancow Wlkp. 72, 70-111 Szczecin, Poland

## Abstract

We investigated effects of bone marrow-derived, lineage-negative cell (Lin^−^BMC) transplantation in acute retinal injury. Lin^−^BMCs were intravitreally injected into murine eyes at 24 h after NaIO_3_-induced injury. Morphology, function, and expression of apoptosis-related genes, including brain-derived neurotrophic factor (BDNF) and its receptor, were assessed in retinas at 7 days, 28 days, and 3 months after transplantation. Moreover, global gene expression at day 7 was analyzed by RNA arrays. We observed that Lin^−^BMCs integrated into outer retinal layers improving morphological retinal structure and induced molecular changes such as downregulation of proapoptotic *caspase-3* gene, a decrease in *BAX/BCL-2* gene ratio, and significant elevation of *BDNF* expression. Furthermore, transplanted Lin^−^BMCs differentiated locally into cells with a macrophage-like phenotype. Finally, Lin^−^BMCs treatment was associated with generation of two distinct transcriptomic patterns. The first relates to downregulated genes associated with regulation of neuron cell death and apoptosis, response to oxidative stress/hypoxia and external stimuli, and negative regulation of cell proliferation. The second relates to upregulated genes associated with neurological system processes and sensory perception. Collectively, our data demonstrate that transplanted Lin^−^BMCs exert neuroprotective function against acute retinal injury and this effect may be associated with their antiapoptotic properties and ability to express neurotrophic factors.

## 1. Introduction

Visual impairment associated with photoreceptor degeneration is a largely untreatable condition affecting millions of people worldwide [1]. Cellular therapies offer an attractive alternative for the treatment of retinal degeneration. Human bone marrow-derived cells (BMCs), enriched in adult stem and progenitor cells (SPCs), present particular advantages for interventional therapy to the eye because they can be directly obtained from the patient by an effortless collection procedure. Consequently, BMCs are nonimmunogenic, which thereby eliminates potential complications associated with immune rejection of allogeneic tissues [2].

The reported beneficial effects of BMCs-based therapies may depend on the trophic activity of SPCs producing various cytokines, including growth factors and extracellular matrix compounds, which regulate the growth, differentiation, and survival of different types of cells [3]. In the study performed by Sensebe et al. it was observed that BMC-induced neuroprotection involves anti-inflammatory and immunomodulatory effects and that neurotrophic factors act through paracrine and/or autocrine interactions between transplanted BMCs and the retinal microenvironment [4]. Moreover, BMCs are known to express several neurotrophins (NTs), including brain-derived neurotrophic factor (BDNF), which can protect injured retinas [5]. BDNF is one of the most studied and promising growth factors for neuronal regenerative therapy and regulates a number of neuronal functions including survival, neurogenesis, and the synaptic plasticity of neurons [6]. It is a 14-kDa neuroprotective protein that preferentially binds to the high affinity TrkB receptor [7]. Supplemental delivery of BDNF in different animal models has been shown to have beneficial effects on the preservation of the structure and function of injured retinas, and BDNF is known to be instrumental in photoreceptor survival [8, 9]. It has been demonstrated that hematopoietic cells secrete bioactive BDNF* in vitro* and support neuronal survival [10]. Recently, Zhang and Wang demonstrated that the subretinal injection of BMCs offered retinal protection by inhibiting apoptosis in a light damage model via the production of BDNF [11].

Despite the encouraging results reported, some unresolved questions remain regarding the optimal cell population that should be used to provide the best neuroprotective outcome of* in vivo* transplantation. Adult bone marrow is known to contain a diverse population of cells that can be divided into lineage-positive (Lin^+^) and lineage-negative (Lin^−^) subpopulations defined by their potential to differentiate into specific elements of blood [12]. The Lin^−^ population of bone marrow-derived cells (Lin^−^BMCs) contains a variety of progenitor cells including hematopoietic, endothelial, and mesenchymal lineages [2]. We recently found that NT expression in the Lin^−^ population of umbilical cord blood (UCB) cells was higher than in unsorted nucleated UCB cells. We also demonstrated that conditioned medium from Lin^−^SPCs support neuronal cell proliferation and survival* in vitro* [13]. Therefore, Lin^−^BMCs seem to be promising candidates for cell-based therapeutic strategies.

In light of these favorable results, we sought to explore whether Lin^−^BMC transplantation is beneficial in mouse retinas acutely injured by intravenous injection of sodium iodate at low doses. Hence, we investigated intravitreally transplanted Lin^−^BMC survival, the pattern of their integration, and their possible differentiation after transplantation in injured retinas. We managed to document the successful incorporation of Lin^−^BMCs into damaged retinas and their survival for up to three months when transplanted intravitreally, and we revealed their beneficial effects via enhancing BDNF expression and decreasing apoptosis. Donor cells exhibited spindle-shaped morphology when examined by confocal microscopy and differentiate into macrophage lineage. Finally, we showed that Lin^−^BMC transplantation had a positive impact on the morphological recovery of acutely damaged retinas.

## 2. Materials and Methods

### 2.1. Animals and Experimental Procedures

Pathogen-free 8- to 12-week-old mature male C57BL mice (Polish Academy of Sciences, Wroclaw, Poland) weighing 27–29 g were used in the experiment. Mice were anesthetized, and sodium iodate (Sigma Aldrich, St. Louis, MO, USA) in PBS at a dose of 20 mg/kg body weight was injected intravenously into the retroorbital sinus. To isolate murine bone marrow cells (BMCs), BM was flushed from the femurs and tibias of 12 pathogen-free, 3-4-week-old GFP-transgenic mice (strain: C57BL/6-Tg(ACTB-EGFP)1Osb/J; Jackson Laboratories, Bar Harbor, ME, USA). The obtained BMCs were depleted of mature hematopoietic cells as well as their committed precursors with a Lineage Cell Depletion Kit using the MiniMACS Separator and LS Column according to the manufacturer's protocol (all from Miltenyi Biotec, Auburn, CA, USA). A cell suspension (1 *μ*L) containing approximately 10^5^ Lin^−^BMCs was injected into the vitreous cavity using a 30-gauge needle 24 h after the retinal lesion was induced by sodium iodate administration. Contralateral eyes were injected with an equal volume of PBS, serving as a control and allowing for paired ocular comparisons. Prior to the procedure, the mice were anesthetized with an intraperitoneal injection of ketamine (40 mg/kg) and xylazine (4 mg/kg) (all from Biowet, Puławy, Poland). All animal procedures were performed according to the regulations in the ARVO Statement for the Use of Animals in Ophthalmic and Vision Research and were approved by the local ethics committee.

### 2.2. Flow Cytometry

A single-cell suspension of isolated Lin^−^BMCs was stained using the Mouse Mesenchymal Stromal Cell 4-Color Flow Cytometry Kit (R&D, Minneapolis, MN, USA), containing conjugated antibodies to CD105-FITC, CD29-PE, Sca-1-APC, and CD45-PerCP. To expand the analysis, some of the samples were also stained with the following antibodies: anti-CD90-APC (R&D, Minneapolis, MN, USA), anti-CD34-APC (BioLegends, San Diego, CA, USA), and anti-Sca-1-PE (BD Biosciences Pharmingen, San Diego, USA). The cells were diluted in 100 *μ*L Staining Buffer (R&D, Minneapolis, MN, USA) and incubated with the antibodies for 45 minutes at 2–8°C. Samples stained with the appropriate isotype control were examined in parallel. After washing, cells were resuspended in fresh Staining Buffer and analyzed by fluorescence-activated analyzer LSRII (BD Biosciences, San Jose, CA, USA). At least 10^5^ events were acquired and analyzed using FACS Diva software (BD Biosciences). The number of cells in each population was expressed as the percentage of the total number of events.

### 2.3. Histological and Immunofluorescence Analysis

For cross sections, the eyes were embedded in paraffin, cut into 5-*μ*m-thick sections, and routinely stained with hematoxylin and eosin (Sigma-Aldrich, USA). For immunofluorescence analyses performed by employing sequential stainings, the 5 *μ*m-thick sections were deparaffinized in xylene (2 × 15 min) followed by hydration in solutions with decreasing ethanol concentrations (100, 95, 80, 75, and 50%) and antigen retrieval (20 min of boiling in citrate buffer, pH 6.0). After blocking in 10% normal horse serum, the sections were incubated with the primary antibody, chicken anti-GFP (1 : 1,000) (Abcam, Cambridge, UK) in PBS complemented with 1% BSA, at 4°C overnight. Next, the sections were incubated with secondary antibody for 1 h at room temperature in the dark using goat anti-chicken-Alexa Fluor-488 antibody (1 : 100) or goat anti-chicken-Alexa Fluor-594 (both from Life Technologies, Paisley, UK), depending on the subsequent secondary antibody used for colocalization. For coexpression analysis, slides were additionally incubated with the following primary antibodies: goat anti-PCNA (1 : 100) (SC Biotechnology, cat number sc-9857, Santa Cruz, CA, USA) (LifeSpan Biosciences, Seattle, WA, USA), sheep anti-TrkB (1 : 50) (Novus Biologicals, Littleton, CO, USA), rabbit anti-MAPK (1 : 50) (Cell Signaling Technology, Danvers, MA, USA), rabbit anti-Doublecortin (1 : 50) (Novus Biologicals, Littleton, CO, USA), mouse anti-NeuN (1 : 50) (GenTex, Zeeland, MI, USA), Rex1 (1 : 50) (GenTex, Zeeland, MI, USA), mouse anti-CRALBP (1 : 50) (Novus Biologicals, Littleton, CO, USA), rabbit anti-Crx 1 (1 : 50) (Bioss, Woburn, MA, USA), rabbit anti- casp3 (1 : 50) (LifeSpan Biosciences, Seattle, WA, USA), or Isolectin GS-IB4 conjugated to Alexa Fluor-488 (1 : 100) (Life Technologies, Paisley, UK). The sections were then incubated with a secondary antibody, including donkey anti-goat-Alexa Fluor-647 (1 : 100) (Life Technologies, Paisley, UK), goat anti-rabbit-TR (1 : 100) (Vector Laboratories, Burlingame, CA, USA), goat anti-mouse-Alexa Flur-488 (Life Technologies, Paisley, UK), or donkey anti-sheep-Alexa Fluor-647 (1 : 100) (Life Technologies, Paisley, UK).

For immunofluorescence analysis of isolated Lin^−^BMCs, samples were fixed with 3.7% paraformaldehyde (PFA) and then smeared on the polylysine-coated slides. After permeabilization in 0.5% Tween 20 (Bio-Rad, Hercules, CA, USA) and blocking with 10% normal goat serum, smears were incubated with the primary antibody rabbit anti-BDNF (1 : 100) (Santa Cruz Biotechnology, cat number sc-546, Santa Cruz, CA, USA) at 4°C overnight. Subsequently, incubation with the secondary goat anti-rabbit-TR antibody (1 : 100) (Vector Laboratories, Burlingame, CA, USA) was performed in the dark. Upon termination, all sections were then counterstained with DAPI solution (Thermo Fisher Scientific, Waltham, MA, USA), mounted, and subjected to microscopy analysis using an LSM700 confocal system (Carl Zeiss, Jena, Germany). At least five eye tissue sections, derived from different parts of retinas of independent five animals/time point of the experiment, were analyzed.

### 2.4. Western Blot Analysis

For western blot analysis, proteins were extracted from retinas at 7, 18, and 28 days and 3 months after sodium iodate administration (5 mice per time point) using the PARIS kit (Life Technologies, Paisley, UK), following the manufacturer's instructions with some modifications. Briefly, retinas were homogenized in the Cell Disruption Buffer containing protease and phosphatase inhibitors (10 *μ*g/mL leupeptin, 10 *μ*g/mL aprotinin, 1 *μ*g/mL pepstatin A, 1 mM sodium fluoride, and 2 mM Na_3_VO_4_) (all from Sigma Aldrich, St. Louis, MO, USA). The mixture was centrifuged, supernatants were collected, and protein concentrations were determined using the Bradford protein assay (Sigma Aldrich, St. Louis, MO, USA). Equal amounts of proteins (20 *μ*g/well) were loaded and separated by 4–20% sodium dodecyl sulfate polyacrylamide gel electrophoresis (SDS-PAGE, mini-PROTEAN II electrophoresis system, Bio-Rad, Hercules, USA) and then transferred to a 0.2-*μ*m polyvinylidene fluoride (PVDF) membrane (Bio-Rad, Hercules, CA, USA). The membrane was probed with primary monoclonal/polyclonal IgG antibodies directed against the amino acid sequences of BDNF, PCNA, and unphosphorylated and phosphorylated p44/42 MAPK (Erk1/2) as follows: rabbit anti-BDNF polyclonal antibody (at 1 : 375 dilution, Santa Cruz Biotechnology, cat number sc-546, Santa Cruz, CA, USA), goat anti-PCNA polyclonal antibody (at 1 : 667 dilution, Santa Cruz Biotechnology, cat number sc-9857, Santa Cruz, CA, USA), rabbit anti-p44/42 MAPK (Erk1/2) monoclonal antibody (at 1 : 667 dilution, Cell Signaling, USA), rabbit anti-phospho-p44/p42 MAPK (Erk1/2) (Thr202/Tyr204) monoclonal antibody (at 1 : 1000 dilution, Cell Signaling), and rabbit anti-casp3 polyclonal antibody (at 1 : 667 dilution, Santa Cruz Biotechnology, cat number sc-7148, Santa Cruz, CA, USA). Immunoreactive bands were detected using horseradish peroxidase-conjugated goat anti-rabbit and donkey-anti-goat secondary antibodies (Santa Cruz Biotechnology, Santa Cruz, CA, USA). Chemiluminescence detection was performed using the ECL Select Detection Kit (GE Healthcare, formerly Amersham Life Sciences, Little Chalfont, UK), and bands were subsequently visualized using a UVP camera (Gel DOC-It Imaging system; Bio-Rad, Hercules, CA, USA). Equal loading in the lanes was evaluated by stripping the blots for 2 h at 37°C and then overnight at room temperature (IgG Elution Buffer; Thermo Fisher Scientific, Waltham, MA, USA). Reprobing was then performed in an analogous manner with a mouse anti-GAPDH monoclonal antibody (Santa Cruz Biotechnology, cat number sc-20357, Santa Cruz, CA, USA) at a 1 : 1000 dilution, followed by an HRP-conjugated secondary antibody as described above. Protein levels were analyzed densitometrically in ImageJ and expressed as the relative density in the right eye (experimentally treated retina) versus the left eye (control retina), which was arbitrarily set to 1.

### 2.5. Real-Time Reverse Transcriptase-Polymerase Chain Reaction (qRT-PCR)

To analyze the mRNA levels for* BDNF*,* TRK2*,* BAX*,* BCL2*, and* CASP3*, total RNA was isolated from retinas at 7, 18, and 28 days and 3 months after sodium iodate administration (5 mice per time point) using the PARIS Kit (Life Technologies, Paisley, UK). To compare mRNA levels of* BDNF* in whole murine bone marrow cells and an enriched population of lineage negative cells, total RNA was isolated using an RNAmini kit (Qiagen, Hilden, Germany). The RNA was reverse-transcribed using the First Strand cDNA Synthesis Kit (Thermo Fisher Scientific, formerly Fermentas International Inc., Waltham, MA, USA). The quantitative assessment of mRNA levels was performed using a Bio-Rad CFX96 Real-Time PCR Detection System (Bio-Rad, Hercules, CA, USA). A 15-*μ*L reaction mixture containing 7.5 *μ*L iQ SYBR Green SuperMix (Bio-Rad, Hercules, CA, USA), 10 ng of complementary (c)DNA template, and 0.9 *μ*M of the following primers:* BDNF* (forward (f): 5′-GCACTGGAACTCGCAATGC-3′, reverse (r): 5′-GTAAGGGCCCGAACATACGA-3′),* TRK2* (f: 5′-CTGGGGCTTATGCCTGCTG-3′, r: 5;-AGGCTCAGTACACCAAATCCTA-3′),* BAX* (f: 5′-TGAAGACAGGGGCCTTTTTG-3′, r: 5;-AATTCGCCGGAGACACTCG-3′),* BCL2* (f: 5′-GTCGCTACCGTCGTGACTTC-3′, r: 5;-CAGACATGCACCTACCCAGC-3′), and* GAPDH* (f: 5′-AGGTCGGTGTGAACGGATTTG-3′, r: 5;-TGTAGACCATGTAGTTGAGGTCA-3′). The cDNA was amplified under the following conditions: 1 cycle at 95°C for 3 min followed by 45 cycles at 95°C for 10 s, 60°C for 15 s, and 72°C for 25 s. Relative quantification of mRNA expression was calculated using the comparative Ct method. Relative quantification (ΔΔCt) of mRNA expression was normalized to the reference gene and calculated using Bio-Rad CFX Manager, Gene Study software (Bio-Rad, Hercules, CA, USA).

### 2.6. Electroretinography

Scotopic and photopic ERGs were recorded 3 months after sodium iodate administration. Following overnight dark adaptation, mice were anesthetized with an intraperitoneal injection of ketamine (40 mg/kg) and xylazine (4 mg/kg). Then, the cornea was anesthetized (Alcaine; Alcon), and the pupils were dilated with 1% atropine. Retinal responses were recorded with gold ring contact electrodes (LKC Technologies, Gaithersburg, USA). Needle electrodes placed under the scalp between the eyes and in the tail served as the reference and ground leads, respectively. ERGs were differentially amplified (0.05–1500 Hz), averaged, and stored using an LKC UTAS BigShot system. ERGs were recorded in response to strobe flash stimuli presented in the LKC Ganzfeld bowl in a protocol similar to those used for human testing. For the assessment of rod photoreceptor function, a strobe white-flash stimulus was presented to the dark-adapted dilated eye with a low flash intensity (24 dB attenuation), and 8 responses recorded at intervals of 8 s were computer-averaged. Mixed rod and cone responses were obtained using stimulation with white flashes of maximum intensity equal to approximately 1.6 cd∗s/m^2^ (Standard Flash, SF, 0 dB attenuation). The retinal responses were measured twice with a 28-s interstimulus interval and averaged. To evaluate the function of cone photoreceptors, animals were light-adapted for 10 min under a white background (32 cd/m^2^). Next, a strobe white-flash stimulus was presented to the dilated eye in the Ganzfeld bowl using the maximum flash intensity (0 dB attenuation), and responses to 8 flashes with an interstimulus interval of 1 s were recorded and averaged. The amplitude of the b-wave was measured from the a-wave trough to the peak of the b-wave or, if an a-wave was not present, from the prestimulus baseline to the peak of the b-wave.

### 2.7. SD-OCT Imaging

To perform the imaging procedures, the pupils of the mice were dilated with 1% atropine. Artificial tears were used throughout the procedure to maintain corneal clarity. Mice were anesthetized with an intraperitoneal injection of ketamine (40 mg/kg) and xylazine (4 mg/kg). Ultra-high-resolution spectral-domain optical coherence tomography (SD-OCT) images were obtained using the Envisu R2200-HR SD-OCT device (Bioptigen, Durham, NC) with the reference arm placed at approximately 1185 mm. SD-OCT images of a specific region of each eye were taken using the optic nerve head as a landmark. Rectangular scans (1.4 mm width, 1000 a-scans/b-scan × 100 frames/b-scan) were obtained while centered on the optic nerve and again with the nerve displaced either temporally/nasally or superiorly/inferiorly.

### 2.8. Statistical Methods

The significance of differences between experimental groups was assessed with the Kruskal-Wallis test followed by the Mann-Whitney test. *P* < 0.05 was considered statistically significant. The data are presented as the mean ± standard deviation (SD). Fisher's exact test was applied for overrepresentation of selected genes in GO biological categories.

## 3. Results

### 3.1. Identification and Characterization of Transplanted BM-Derived Lin^−^ Cells* In Vitro*

**Figure 1:**
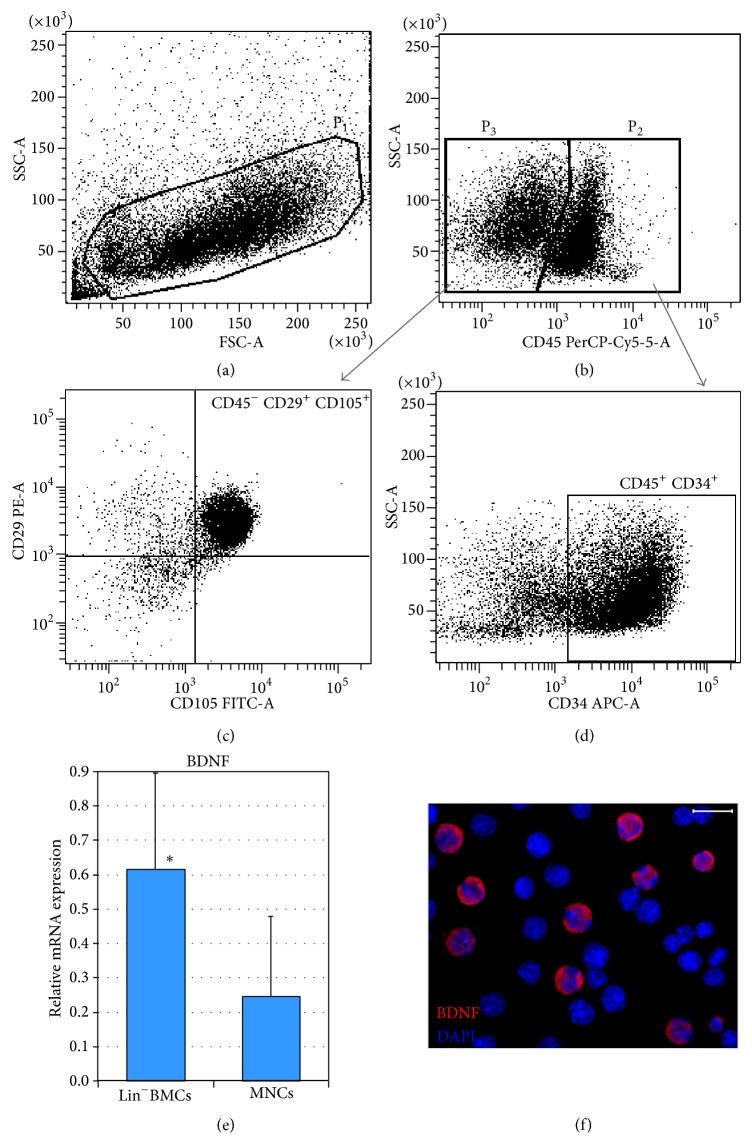
Quantification of the hematopoietic and mesenchymal origin of a BM-derived Lin^−^ cell population by flow cytometry. Lin^−^BMC distribution based on FSC (Forward Scatter) and SSC (Side Scatter) parameters that describe their size and granularity, respectively (a). The cells enclosed in region P1 were further subdivided into hematopoietic (region P2) or nonhematopoietic (region P3) cell populations based on their CD45 antigen expression (b). CD45^−^ BMCs were additionally analyzed based on their CD29 and CD105 antigen expression to determine their mesenchymal origin (c). Similarly, CD45^+^ BMCs were analyzed according to their CD34 antigen expression to exclude mature hematopoietic cells and to determine the number of hematopoietic stem/progenitor cells (d). Expression of mRNA for* BDNF* in isolated Lin^−^BMCs and BM-derived mononuclear cells (e). Immunocytofluorescence image of Lin^−^BMCs depicts the expression of BDNF protein (red). Nuclei are visualized with DAPI staining (blue) (f). Scale bar = 20 *μ*m. ^*^ *P* < 0.05.

The phenotype of immunomagnetically isolated lineage-negative BM cell population was verified by fluorescence activated cell sorting (FACS) analysis before the injection procedure. This analysis revealed that 53.5 ± 4.8% of Lin^−^BMCs were positive for CD34 antigen and that 16.8 ± 6.7% of Lin^−^BMCs were positive for Sca-1. Moreover, 42.4 ± 12.5% of the analyzed cells were CD90 positive, and 74.3 ± 9.3% of Lin^−^BMCs were CD45 positive. Similarly, to determine the expression of mesenchymal- and endothelial-associated surface antigens on transplanted Lin^−^BMCs, a FACS evaluation was performed. Accordingly, the CD29 antigen was expressed in 58.3 ± 5.9% of isolated Lin^−^BMCs, and CD105 was expressed in 36.4 ± 12.3% of analyzed cells. Coexpression analysis revealed that almost half (49.8 ± 7.2%) of the transplanted cells coexpressed both antigens (CD34^+^/CD45^+^) and that 23.7 ± 10.2% cells presented a CD29^+^/CD105^+^/CD45^−^ phenotype (Figures 1(a)–1(d)). These findings indicate that Lin^−^BMCs are a heterogeneous population of cells that express different markers, which are mostly characteristic of the hematopoietic and mesenchymal lineages.

Prior to the intravitreal transplantation of Lin^−^BMCs, we also analyzed the mRNA expression for BDNF, and we found that these cells abundantly expressed this particular neurotrophin when compared with BM-derived mononuclear cells (MNCs) (Figure 1(e)). In addition, we determined the expression of BDNF protein in Lin^−^BMCs by immunofluorescence staining and visualized it using confocal microscopy. Thus, we corroborated the data indicating that isolated Lin^−^BMCs actively expressed BDNF protein as shown in Figure 1(f). Taken together, these results demonstrate the constitutive expression of BDNF in lineage-negative BM cell population and suggest that these cells might induce neuroprotection in retinal tissue upon intravitreal transplantation in a paracrine manner.

### 3.2. Homing, Migration, and Differentiation of Transplanted GFP^+^Lin^−^BMCs within Injured Retina

**Figure 2:**
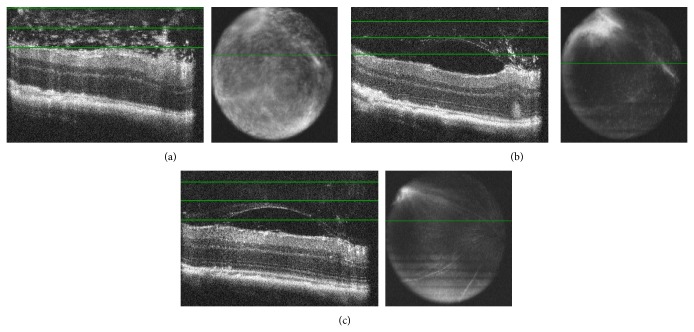
*In vivo* monitoring of injected cell in damaged retinal tissue. A representative SD-OCT image of an injured retina on the 7th day after Lin^−^BMC transplantation (a). The green lines on the OCT images (left side) indicate the boundaries of tissue depth displayed in an* en face* two-dimensional fundus projection (right side). The depth-dependent fundus image at this time point shows injected cells in the vitreous. The cells were uniformly distributed and were homogenous in appearance. The SD-OCT analyses on the 28th day (b) and the 3rd month (c) showed the absence of the injected Lin^−^BMCs in the vitreous cavity.

We sought to determine if Lin^−^BMCs could be efficiently delivered to the retina through intravitreal pars plana injection and, once there, if they could be successfully incorporated into injured retinal tissues. In particular, our goal was to assess the kinetics of Lin^−^BMCs trafficking from the vitreous chamber and cell integration within the damaged retina. For this reason, we monitored the injected eyes repeatedly in each mouse using optical coherence tomography (SD-OCT). On the 7th day after transplantation, depth-selective SD-OCT fundus images of the vitreous gel revealed hyperreflective opacities in the vitreous that likely represent injected cells. The opacities were uniformly distributed in the vitreous body and had a homogenous appearance (Figure 2(a)). By day 28, the hyperreflective regions in the vitreous gel disappeared (Figure 2(b)). In the 3rd month after injury,* en face* projection focused on the vitreous body showed that the injected cells were absent in the vitreous cavity, and an exact morphology of the vitreous gel was observed that may indicate the effective migration of transplanted cells toward the retinal tissue injury (Figure 2(c)).

**Figure 3:**
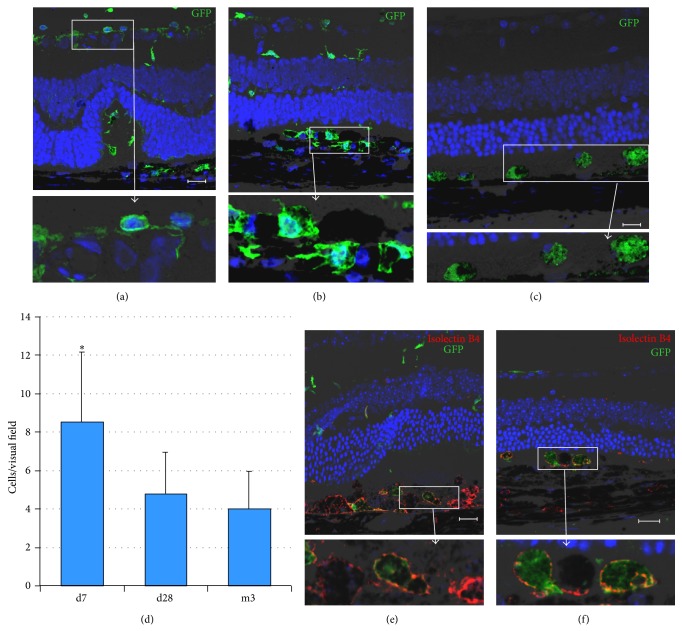
Longitudinal follow-up of the fate of Lin^−^BMCs at different time points post intravitreal transplantation. Immunofluorescence analysis of retinas after intravitreal GFP^+^Lin^−^BMCs delivery showed transplanted cells (green) on the 7th day (a, b) and at 3 months after injection (c). Quantitative analysis of the total number of incorporated Lin^−^BMCs per eye section (*n* = 5/time point) (d). Coreactivity of GFP (green) and Isolectin-B4 (red) by fluorescent lectin staining showed that transplanted GFP^+^Lin^−^BMCs locally differentiated into Isolectin-B4-positive macrophages (inserts) in Lin^−^BMC-injected eyes on the 7th day after transplantation (e) and at 3 months after injection (f). Scale bar = 20 *μ*m. ^*^ *P* < 0.05 for day 7 versus other time points.

Next, we evaluated the localization of transplanted Lin^−^BMCs expressing GFP protein (GFP^+^Lin^−^BMCs) in the damaged retina by histological section examination. We found that the transplanted cells were incorporated into the retinal pigment epithelium (RPE) and photoreceptor retinal layers, where they survived up to 3 months (Figures 3(a)–3(c)). To the contrary, we did not observe any homing of transplanted cells into the control, uninjured retinas (data not shown). Interestingly, the transplanted GFP^+^BMCs were incorporated in a time-dependent manner, and the greatest number of cells was detected at day 7 after injury with a gradual decrease continuing up to the 3rd month after injury. A quantitative analysis of the total number of incorporated GFP^+^Lin^−^BMCs per eye section is summarized in Figure 3(d). We also observed that the homing pattern of donor GFP^+^ cells was notably different between the early and late time points, after cell transplantation. A widespread migration was observed on the 7th day, with a characteristic alignment of spindle shape cells in lateral fashion along the inner nuclear layer (INL) of the retina (Figure 3(a)). Additionally, at this time point, the transplanted cells were found to be clustered in the deeper retinal layers at the injury site and possessed the ramified morphology (Figure 3(b)). On the contrary, at the 3rd month after injury, the vast majority of the GFP^+^Lin^−^BMCs were amorphous/oval in shape and found to be aligned along the RPE-photoreceptor junction (Figure 3(c)).

Finally, to evaluate whether transplanted Lin^−^BMCs could differentiate into other retinal cell types, the immunofluorescence analysis directed against photoreceptors (Crx1), neuronal markers (NeuN, Doublecortin, Pax2), and glial cell markers (GFAP, CRALBP) was performed. As a result, no GFP^+^ cells localized intraretinally or subretinally were positively stained for any of the molecular markers at the examined time points (data not shown). These results may indicate that there is a lack of intermediate differentiation stages in transplanted GFP^+^Lin^−^BMCs expressing neuronal and photoreceptor markers concurrently. On the other hand, we observed that the transplanted GFP^+^ cells coexpress isolectin B4, which is a microglial and macrophage marker (Figures 3(e) and 3(f)). More importantly, we realized that GFP^+^Lin^−^BMCs positive for IsolectinB4 were localized predominantly at the site of injury (RPE/photoreceptor junction). This may indicate that the existence of very specific conditions (e.g., neuronal/epithelial cell apoptosis or retinal cell destruction) is required for BMC differentiation, most likely due to the presence of strong endogenous stimuli for cell debris clearance at that site.

### 3.3. Neuroprotective Effects of Transplanted Lin^−^BMCs

**Figure 4:**
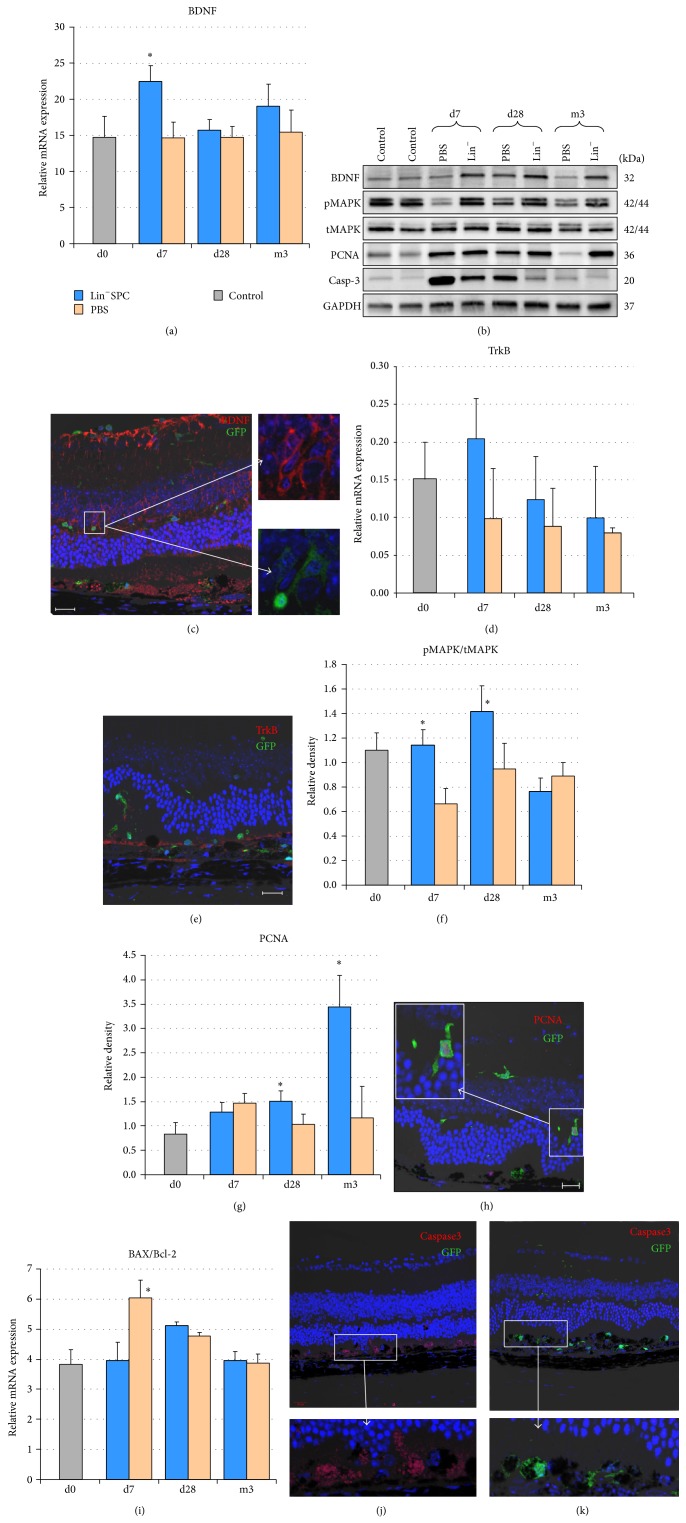
Molecular analyses of potential* in vivo* effects of Lin^−^BMCs on injured retinas at different time points post injury.* BDNF* mRNA (a) and BDNF protein (b-upper panel) expression time course analyses show a significant increase in Lin^−^BMC-treated eyes compared to PBS-injected eyes on the 7th day after transplantation. Immunoblotting for the BDNF, pMAPK, tMAPK, PCNA, and active caspase-3 of retinal lysates collected from control, uninjured animals (Control), PBS injected and Lin^−^BMC-treated mice on d7, d28, and m3 after injury (b). Double-stained retinal sections (anti-BDNF and anti-GFP) were used to visualize the coexpression of BDNF and GFP cell markers in transplanted Lin^−^BMCs (insert) (c). Quantitative PCR analysis for relative quantification of* TRK-B* mRNA displayed a markedly increased level on the 7th day after transplantation (d). Representative immunofluorescence for Trk-B expression following Lin^−^BMC transplantation revealed that its expression is mostly restricted to RPE/photoreceptor junction (e). The western blot analysis and densitometry for relative protein quantification of the active, phosphorylated form of p44/42 MAPK (Erk1/2) revealed its markedly increased expression on d7 and d28 in the Lin^−^BMC-transplanted retinas (f). The western blot analysis and densitometry for relative PCNA quantification demonstrated a strong overexpression of the protein in the retinas collected from the Lin^−^BMC-treated mice on d28 and m3 after injury (g). Mean values ± SDs are presented in the diagrams. Double-stained sections (anti-GFP and anti-PCNA) were used to visualize the location of proliferating Lin^−^BMCs (insert) (h). Quantitative PCR analysis of* BAX*/*BCL-2* ratio following Lin^−^BMC transplantation (i) (*n* = 5/time point). There were significant reductions in the* BAX*/*BCL-2* ratio on d7 after Lin^−^SPC injection. The immunofluorescent localization of active caspase-3 demonstrated that its expression was detected selectively in the PBS-injected, control retinas at the site of injury (j, k). The scale bar = 20 *μ*m. ^*^ *P* < 0.05.

Having demonstrated that transplanted GFP^+^Lin^−^BMCs could home the sites of retinal injury and integrate within damaged retinal layers, we sought to determine whether these cells provide enhanced and extended trophic support for the reparative processes in injured retinas. Consequently, we performed quantitative PCR and Western blot analysis to examine the expression of BDNF in injured retinas at selected time points after Lin^−^BMCs transplantation. We found that such transplantation procedure induced a significant increase of mRNA expression for* BDNF* on the 7th day after injection (Figure 4(a)). Furthermore, these differences, observed at selected time points, in* BDNF* expression were corroborated by Western blot analysis (Figure 4(b)). Interestingly, during the long-term observation through the 3rd month after transplantation,* BDNF* protein expression level was also considerably increased in BMC-treated eyes compared to contralateral PBS-treated control eyes (Figure 4(b)). Next, the cellular origin of BDNF expression was confirmed by our anti-BDNF immunofluorescence study. We found that BDNF was persistently expressed by GFP^+^ cells, although not exclusively (Figure 4(c)). These data suggest that transplanted Lin^−^BMCs could provide an intrinsically continuous supply of BDNF for injured retinas for at least 3 months after transplantation. Moreover, we found slightly increased* TRK-B* mRNA levels at early time points after Lin^−^BMC transplantation, but these changes were not statistically significant (Figure 4(d)). Additionally, to determine the precise localization of the BDNF-target cells, we performed immunofluorescence analysis of retinas collected from Lin^−^BMCs-treated mice using an anti-TrkB antibody. We found that TrkB expression in the retina after Lin^−^BMC transplantation was limited to the RPE/photoreceptor junction (Figure 4(e)). These results show that the key activity of BDNF occurs at the site of injury.

Subsequently, to determine whether Lin^−^BMC transplantation could stimulate local tissue repair and activate prosurvival and proproliferative signal transduction pathways in the retinal cells, we analyzed the expression of the selected survival-related proteins, such as mitogen-activating protein kinase (MAPK-Erk1/2) in phosphorylated (pMAPK) and constitutive total (tMAPK) forms, as well as proliferating cell nuclear antigen (PCNA). Accordingly, we performed western blot analysis of lysates isolated from injured retinas harvested on day 7, day 28, and 3 months after sodium iodate administration and Lin^−^BMC transplantation. As shown in Figure 4(b), in the analyzed tissues, we observed stable expression of tMAPK throughout the experimental period in both eyes. Moreover, a significant overexpression of pMAPK in the Lin^−^BMC-treated eyes compared with the contralateral control eyes was observed, which was initiated on the 7th day and continued in a sustained fashion until the 28th day (Figure 4(f)). This finding might indicate that Lin^−^BMC transplantation triggered intracellular mechanisms that are essential for the process of endogenous repair, as MAPK is a key regulator of cell proliferation, differentiation, mitosis, and cell survival, among many other cell functions. Likewise, the expression of PCNA was significantly increased at the 28th day and in the 3rd month in the BMC-treated eyes compared with the contralateral control eyes (Figures 4(b) and 4(g)), indicating increased proliferative and proregenerative activity of retinal cells particularly supported by Lin^−^BMC transplantation. In addition, to assess the proliferative potential of the transplanted GFP^+^Lin^−^BMCs, we performed immunohistochemistry against PCNA, and we found several proliferating PCNA^+^ cells among migrating GFP^+^ cells in the retinal INL (Figure 4(h)).

Finally, to determine whether the neuroprotective effects of Lin^−^BMCs were associated with decreased apoptosis in injured retinal cells* in vivo*, the apoptotic process was assessed in the retinas collected at different time points after transplantation. Apoptosis is tightly controlled by the balance between pro- and antiapoptotic members of the Bcl-2 protein family. Changes in the relative expression levels of such molecules will ultimately decide the cell fate [14]. Therefore, we determined the mRNA expression level for the proapoptotic gene* BAX* and the antiapoptotic gene* BCL-2* by quantitative PCR. We found that Lin^−^BMC treatment led to a significant decrease in the* BAX/BCL-2* expression ratio in the early time point after injection (Figure 4(i)). Accordingly, we used western blot analysis of the retinal lysates to compare the caspase-3 activation in injured retinas, and we found that Lin^−^BMCs transplantation significantly decreased the expression of its cleaved products in retinal tissue, especially at early time points such as day 7 after injury (Figure 4(b)). To further confirm our results, we performed an immunofluorescence analysis of the retinas collected at this early time point using an anti-caspase-3 antibody. Accordingly, we revealed that the contralateral/control retinal tissue evidently showed positive immunostaining at the RPE/photoreceptor junction (Figure 4(j)), while a negative reaction was found within the Lin^−^BMC-treated retinas (Figure 4(k)). These results would indicate that Lin^−^BMC treatment may potentially improve/enhance the autocrine and paracrine signaling in injured retinas, which reduces apoptosis of endogenous cellular components of murine retina and, most likely, in transplanted Lin^−^BMC that survived long term in sites of retinal damage.

### 3.4. Intravitreal Injection of Lin^−^BMCs Enhances Retinal Morphological Recovery after Injury

**Figure 5:**
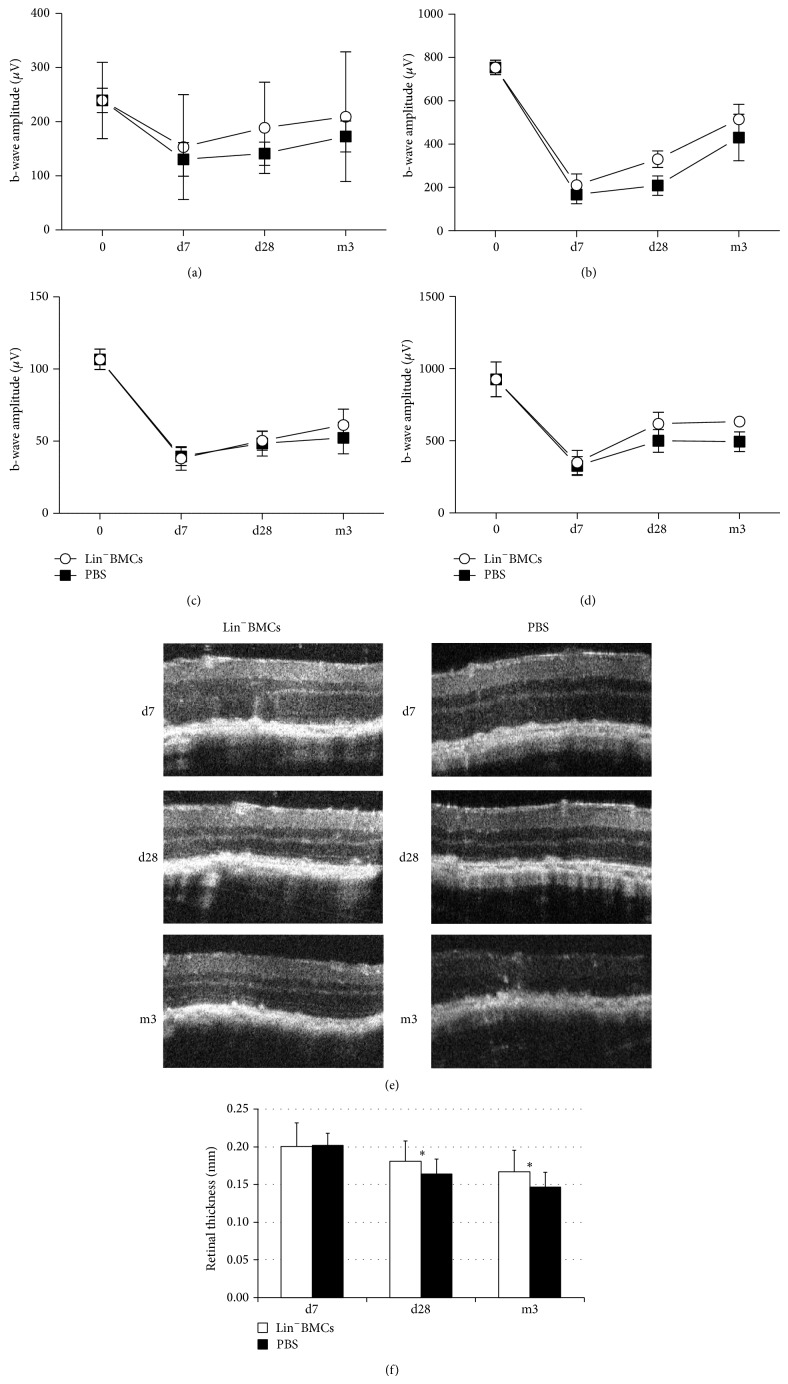
Effects of Lin^−^BMC transplantation on retinal morphology and the b-wave ERG amplitudes over time. The scotopic (a), mixed rod-cone (b), and photopic (c) response, as well as oscillatory potentials (d), were analyzed repeatedly on the same individual mice during the time of the experiment. The results are presented as the mean ± SD (*n* = 5). Representative* in vivo* SD-OCT images of the retina on the 7th and 28th day and at the 3rd month after injury showing a considerable improvement in the architecture of the outer and inner photoreceptor segments of the right eyes treated with Lin^−^BMCs (e). Quantitative comparative analysis of the total retinal thickness of the Lin^−^BMC-treated retinas compared with the control eyes (f). ^*^ *P* < 0.05 for the right eyes treated with Lin^−^BMCs versus the left eyes injected with PBS (*n* = 5).

To assess the biologic effects of lineage-negative BM cell transplantation on physiologic retinal function, we monitored the bioelectrical retinal response after sodium iodate administration at the 7th day, 28th day, and 3-month time points. Electroretinography (ERG) revealed a long-term preservation of retinal electrical activity in eyes that received Lin^−^BMC transplantation compared to control eyes that received PBS injection. The changes in scotopic and photopic ERGs recorded from the examined animals at different time points after NaIO_3_ injection and cell therapy are summarized in Figure 5. The ERG recording analysis revealed a substantial reduction of the b-wave amplitude on the 7th day after transplantation in both groups of eyes (grafted with BMCs and control). Importantly, reduction of more than half of the b-wave amplitude is indicative of a considerable loss of photoreceptor function after NaIO_3_ injection and was consistent with our previous observations (Machalińska et al., 2013). Nevertheless, we cannot exclude the possibility that vitreous opacities would attenuate the ERG response at this selected time point.

Remarkably, after 28th day after injury, the physiological rescue became more pronounced in the Lin^−^BMC-treated eyes compared to the control eyes. Similarly, at the 3rd month after injury, we could see a moderate increase of b-wave amplitudes in BMC-treated eyes compared to control eyes. However, the difference in b-wave amplitudes between the two groups of eyes was not significant. These results may indicate that transplanted Lin^−^BMCs might have some positive influence on the electrophysiological function of injured retinal tissues.

Consequently, to determine whether the therapeutic intervention affected the retinal structure, we used the OCT technique to follow the retinal morphology at selected time points after NaIO_3_ administration and Lin^−^BMC transplantation (Figure 5(e)). At the 3rd month after injury, the OCT images obtained from Lin^−^BMC-treated eyes showed complete restoration of the laminated organization of the outer retina at the site of injury, indicating that the photoreceptors regained their morphology. By contrast, in the contralateral control eyes, which were injected with PBS, we detected the disintegration of the outer retinal zone architecture. A quantitative comparative analysis of the total retinal thickness of the Lin^−^BMC-treated retinas compared with the control eyes is summarized in Figure 5(f). Beginning on the 28th day, the control retinas demonstrated a slow and progressive decline in their total thickness, which continued to decrease significantly until the third month of the experiment, compared with the Lin^−^BMC-treated eyes. This finding might indicate that the processes of cell death are active in the control retinas, causing a continuous decrease in the number of retinal cells and subsequent disintegration of the retinal tissue. By contrast, the intravitreal transplantation of Lin^−^BMCs markedly ameliorated the morphological structure of the injured retina.

### 3.5. Differential Gene Expression Profile in Retinas Treated with Intravitreal Transplantation of Lin^−^BMCs

To further understand the molecular mechanisms underlying the cytoprotective effect of Lin^−^BMC transplantation to the acutely injured retinas, a comparative RNA microarray analysis was performed to investigate the differences in the global gene expression profiles on the 7th day after NaIO_3_ injury between retinas from eyes treated with Lin^−^BMCs and control PBS-injected eyes. In total, 1458 genes were identified that showed at least a 4-fold differential regulation in the analyzed retinas. Among them, 1364 genes were found to be downregulated between 4- and 37-fold upon Lin^−^BMC transplantation; representative examples are listed in Table S1 (see Supplementary Material available online at http://dx.doi.org/10.1155/2015/620364). Interestingly, the lowest expression level (37-fold decrease) was observed for the* COPA* gene, which is required for intracellular protein transport between the endoplasmic reticulum and the Golgi compartment; thus, it is essential for protein secretion and influences the structural integrity of cellular Golgi network. Based on an unbiased examination of the Gene Ontology (GO) classification of Biological Process, the 1364 downregulated genes were located in 152 major ontologies (*P* < 0.05), which included the following: transport (356 genes); negative regulation of biological process (283 genes); cellular catabolic process (196 genes); programmed cell death (149 genes); negative regulation of metabolic process (138 genes); negative regulation of macromolecule metabolic process (129 genes); negative regulation of cellular metabolic process (127 genes); regulation of catalytic activity (127 genes); regulation of cell death (123 genes); cellular response to chemical stimulus (102 genes); cellular response to stress (83 genes); regulation of response to stress (55 genes); negative regulation of cell proliferation (46 genes), response to oxidative stress (29 genes), and neuron death (22 genes).

**Figure 6:**
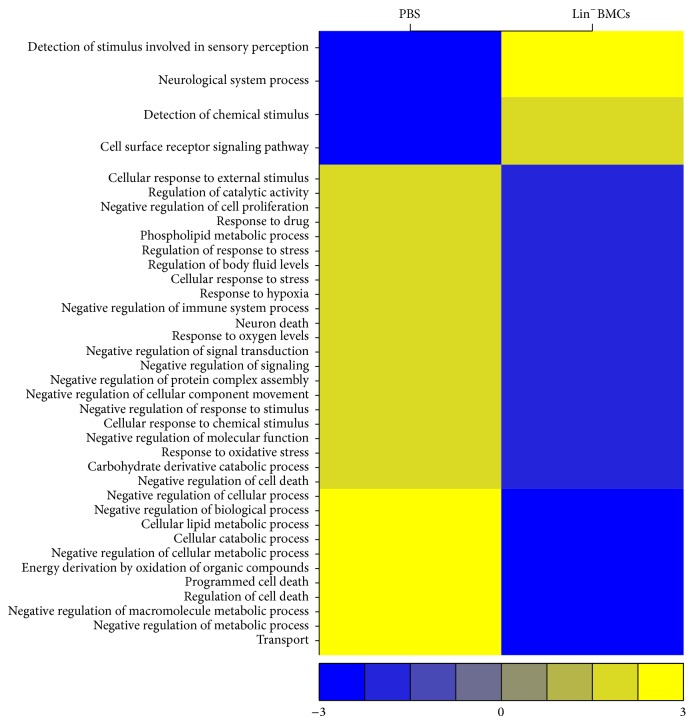
Global changes in gene expression in murine retinas on the 7th day after NaIO_3_-injury. The heat-map represents expression levels of genes representing particular gene ontologies (GOs) with large gene expression changes (fold change > 4) in Lin^−^BMC-treated eyes compared to PBS-injected control eyes. For a given GO, an average value was computed and subtracted from each observation. Each column comprises a set of horizontal lines, with each line representing a particular GO. Gene expression levels comprising a particular GO are indicated on a color scale, with yellow corresponding to the highest level of expression and blue corresponding to the lowest level; the range of expression rate from the analyzed genes is shown below the graph. The GO terms, listed on the left side of the graph (*y*-direction), were selected if the genes annotated with the corresponding GO terms were considered statistically significantly overrepresented.

Apart from the downregulated genes, 94 genes were upregulated (4- to 38-fold). Table S2 illustrates a selection of the upregulated genes. Importantly, the highest expression level was observed for the* TMEM107* gene, which is required for normal Sonic hedgehog (Shh) signaling in neural development. Moreover, the analysis identified significant increases (at least a 4-fold change) in the expression of olfactory receptor (OR) gene superfamily, represented by 25* OLFR* genes encoding different members of OR family. Among the upregulated genes, GO analysis revealed several functional ontologies (*P* < 0.01), including the following: cell surface receptor signaling pathway (30 genes); detection of chemical stimulus (24 genes); neurological system process (24 genes); and detection of stimulus involved in sensory perception (24 genes). A summary version of the temporal distribution of genes by Gene Ontology classification of Biological Processes is shown in Figure 6.

All together, the analysis of the global gene expression changes on 7th day after injury in Lin^−^BMC-treated retinas revealed that a large number of gene expression alterations can be categorized into two functional profiles. First, the majority of the downregulated genes govern the cellular pathways associated with neuron cell death, the regulation of cell death and apoptosis, the response to external and chemical stimuli, the response to oxidative stress and hypoxia, and, finally, the negative regulation of cell proliferation. Second, the upregulated genes that were observed at the same time point were associated with signaling pathways involved in sensory perception and neurological system process.

## 4. Discussion

The retina, in contrast to other central nervous system targets, offers numerous benefits that make it favorable for the transplantation of bone marrow-derived stem and progenitor cells. For this reason, there has been considerable research progress related to the use of such cells for transplantation in retinal degeneration as a therapeutic strategy [2]. Because BMCs can be isolated without adverse effects, autologous BMC transplantation is an attractive choice to avoid immune rejection and the ethical concerns of using fetal tissues. Several studies on the migration and differentiation of BMCs into the neural retina have shown that BMCs are integrated into an injured retina after cell transplantation [2, 15]. Likewise, we have observed that BMCs transplanted to the vitreous cavity pass through different retinal layers while directly migrating toward the injury site in the RPE-photoreceptor junction. We managed to document that, on the 7th day after injection, the vast majority of the GFP^+^ cells presented the ramified morphology while passing through the retinal layers. On the contrary, at the 3rd month after injury, the transplanted cells were amorphous/amoeboid in shape and positioned in the subretinal space. Previous works have also reported a similar incorporation pattern of donor cells in mechanical injury and transgenic retinal damage animal models [16, 17]. Retinal injury seems to be essential for stem cell homing/engraftment and differentiation, which is clearly supported by the lack of incorporation of the cells in control eyes without retinal damage observed in our study. Similarly, Chacko and colleagues demonstrated that although ocular stem cells do not incorporate adult retinas or uninjured retinas, they do so when transplanted in animals with retinal injuries [17, 18]. While different strategies of cell administration into the eye (e.g., subretinal transplantation or intravitreal injection) have been considered in the abovementioned studies, the intravitreal technique reveals some important benefits. It is an easy, relatively noninvasive, non-time-consuming, and highly repetitive procedure. The latter is of special importance because further observation and the detailed comparison with control eyes require a standardized and highly reliable protocol of cell administration to the eye. One can expect that intravitreal administration would not be as efficient as subretinal injection for RPE regeneration. However, the transplanted cells possess a strong migratory capacity and can traffic with the chemokine gradient to the damaged tissue [17]. Likewise, in the current study, we observed that Lin^−^BMCs are able to engraft the RPE-photoreceptor junction and may induce the prosurvival and proliferative signals there.

Although the mechanisms of action of Lin^−^BMCs after transplantation are not fully elucidated, they may include (i) differentiation to specialized cells, for example, retinal epithelial cells or neurons, (ii) acting by paracrine or autocrine effects through the secretion of trophic factors, for example, the production of soluble mediators to promote residual cell survival, and, finally, (iii) contribution to immunomodulatory functions [19]. Several studies showed that transplanted BMCs develop morphological and phenotypic neural, glial, and epithelial markers, thus providing evidence for their differentiation into several types of neural and other retinal cells, including photoreceptors, retinal pigment epithelium, and endothelial cells [17, 20–22]. Likewise, Harris et al. have used highly selected BMCs, based on their phenotype (CD133+) as a possible regenerative strategy for the acutely injured retinas [23]. They observed that the transplanted CD133+ BMCs could migrate to the injured RPE layer, could differentiate into cells with significant RPE morphology, and would provide a functional recovery of the visual system. However, there is a growing body of evidence that suggests that such direct differentiation processes should be called into question, as these cells seem to have a very limited ability to directly replace injured retinal tissue components. It has been suggested that the acquired neural-like morphology results from stress-connected artifactual cell overstaining rather than from genuine differentiation [3, 24]. Indeed, in our study, no intravitreally transplanted Lin^−^BMCs were found to replace the lost photoreceptors by differentiation into neural or any other retinal-derived cells. In this respect, it is pertinent to mention the work of Otani et al. who showed the absence of lineage-negative cell-derived neurons in the ONL layer, thus ruling out the possibility that the injected cells directly transform into photoreceptors [25]. However, intravitreally injected cells were documented to stabilize retinal blood vessels, thereby delaying the progression of the degenerative process and supporting cone photoreceptor rescue.

One hypothesis is that locally administered BM-derived SPCs migrate into the retina and secrete neurotrophic factors to promote residual cell survival. The inhibition of photoreceptor apoptosis by neurotrophic factors released from bone marrow mesenchymal stem cells injected subretinally has been reported in previous studies [11]. Our results confirmed that Lin^−^BMC treatment attenuates cell apoptosis in treated retinas via an increase in endogenous antiapoptotic* BCL-2* gene expression and the downregulation of proapoptotic* BAX* gene expression. Consequently, in Lin^−^BMC-transplanted eyes, the apoptosis-associated caspase-3 activation was significantly suppressed. Furthermore, we observed a significant overexpression of* BDNF* mRNA at the 7th day after injury in Lin^−^BMC-transplanted eyes. We also confirmed, using in situ immunofluorescence, that transplanted GFP^+^Lin^−^BMCs were positive for BDNF protein. Subsequently, using a sensitive and specific western-blot analysis, we observed significant MAPK activation in retinal cells exposed to Lin^−^BMC transplantation. Moreover, Lin^−^BMC transplantation considerably increased the proliferation of retinal cells during long-term observation. This finding might be a consequence of increased prosurvival and proregenerative activity of the injured retinal cells that were particularly supported by Lin^−^BMCs. Taken together, these results suggest that the proregenerative effects observed in retinas after Lin^−^BMC transplantation were due to the trophic effects of transplanted cells. We recently demonstrated that neurotrophin expression, including BDNF, in lineage-negative SPCs was robustly increased under stress-related serum-free conditions* in vitro* [13]. In the same notion, the cell isolation procedures from bone marrow and the subsequent therapeutic administration of such these cells into the vitreous cavity, which contains a relatively low level of plasma proteins, might induce metabolic stress in transplanted cells. Hence, we cannot exclude the possibility that the vitreous microenvironment might accelerate the therapeutic potential of BM-derived Lin^−^ cells by increasing the production of neurotrophic factors, thereby augmenting their regenerative potential.

Our data also showed that intravitreal Lin^−^BMC transplantation modulates and downregulates the molecular pathways underlying multiple cellular metabolic processes significantly related to cell death and cellular response to stress stimuli. As we previously reported, the NaIO_3_-induced chemical injury significantly upregulated gene expression pathways associated with cell death, apoptosis, and acute response to stress in the retinas collected on the 7th day after injury induction as compared to control uninjured retinas [26]. Now, we focused on the specific endogenous retinal response to Lin^−^BMC intravitreal administration. The performed analysis of the global gene expression alterations that suggested that the repressed genes mostly included those governing cellular pathways associated with neuron cell death, the regulation of cell death and apoptosis, the response to external and chemical stimuli, the response to oxidative stress and hypoxia, and, finally, the negative regulation of cell proliferation. To the contrary, we managed to demonstrate that Lin^−^BMC therapy was also accompanied by coordinated mRNA upregulation of several members of the olfactory receptor (ORs) family. The OR proteins are members of the class A rhodopsin-like family of G-protein-coupled receptors (GPCR) arising from single coding-exon genes that comprise the largest gene family in the genome, consisting of over 900 genes in humans and 1500 genes in mice [27]. Interestingly, the sensory receptor cells (e.g., olfactory receptors, hair cells in the ear, and photoreceptors in the retina) share several common features of ongoing sensory cell production, including common molecular expression patterns during their developmental period. Moreover, there are great similarities in the developmental mechanisms of different specialized sensory epithelia that are highly relevant to their regenerative capacities [28]. Of the specialized sensory epithelia, the olfactory epithelium shows the most robust regeneration in response to injury [29]. The molecular mechanisms that maintain cell identity are still not very well understood, and further research into the epigenetic response of cells to injury and regeneration is warranted. However, we hypothesize that the injury-related activation of specific signaling pathways that result in OR upregulation may also be stimulated in the retina, as ORs belong to rhodopsin-like family of GPCRs. Nevertheless, the microenvironment of the postnatal retina differs from that which is present during embryonic development. Thus, we were not able to directly observe the differentiation of injected cells into neural or other retinal lineages.

Another possible mechanism of retinal neuroprotection after cell transplantation is that locally administered Lin^−^BMCs differentiate into macrophages and phagocytose cellular debris, thus clearing the degenerative environment. There is previously reported evidence that BMCs exhibit unique migratory and phagocytic properties in damaged retinas. Sasahara et al. demonstrated that BM-derived cells were recruited to the degenerating retina, where they differentiated into microglia, and were subsequently localized to the degenerating neurons [30]. Interestingly, we observed phagocytosed melanin remnants inside GFP^+^ cells and confirmed their macrophage differentiation by in situ immunofluorescence analysis. We suppose that the transplanted BMCs can exhibit phagocytic function and may thus modulate the local inflammatory environment. Interestingly, a recent report on Alzheimer's disease demonstrated that BM-derived microglia can eliminate amyloid deposits by a cell-specific phagocytic mechanism [31]. There is growing evidence that the clearance of cell debris and apoptotic cells can display anti-inflammatory and immunosuppressive effects, and, in contrast, the defective clearance of apoptotic cells may be linked to inflammation and autoimmune diseases [32]. Likewise, in an established mouse model for AMD, the impaired macrophage recruitment resulted in alterations similar to those observed in human AMD [33]. Moreover, strong evidence for this hypothesis comes from the MFG-E8-deficient mouse model bearing a macrophage insufficiency in which the defective removal of apoptotic cells can lead to inflammatory-type diseases [34]. Similarly, there are studies suggesting an important role of macrophages in axonal regeneration, possibly via secretion of neurotrophic factors [35, 36]. This may indicate that neurotrophins locally released by macrophages may act as autocrine or paracrine factors responsible for regulation of inflammatory responses. Similarly, it has been demonstrated that umbilical cord blood mesenchymal stem cells stimulate the injured brain via microglia/macrophage activation and significantly increase BDNF concentration in the lesioned tissue [37]. Moreover, it has been previously reported that in the light-induced retinal degeneration model, microglia secrete different NTs, including nerve growth factor, BDNF, or ciliary neurotrophic factor, and secondarily modulate neurotrophic factor expression in Müller glia, thus contributing to the protection of PR cells [38]. Based on this observation, BMC-derived cells with a microglia/macrophage-like phenotype and the ability to clean up cell debris would play a significant role as modulators of neuronal cell survival and apoptotic cell death and would thus preserve retinal function. It is also possible that in addition to direct NT secretion by BMCs, these cells modulate secondary NT expression in retinal Müller glia, contributing to the protection of photoreceptors. Likewise, we recently demonstrated that the expression of NTs in glial cells increased in response to NaIO_3_-mediated acute retinal injury [26]. Importantly, cone outer segments and ganglion cells normally produce the BDNF protein [39]. Therefore, the physiological expression of BDNF in the retinal cells and its functional increase during retinal injury may augment the final beneficial outcome of the therapeutic strategy using the BDNF-rich Lin^−^BMCs for intravitreal administration.

Notably, in the current study, we found that in the retinas after Lin^−^BMC transplantation, the TrkB expression was limited to the RPE/photoreceptor junction. This result might suggest that the strongest biologic activity of BDNF occurs at the site of injury. The expression of TrkB has been previously reported to be present within the RPE [40]; however, in some studies, this expression also occurs in other retinal tissue zones, including the retinal ganglion cells or cone outer segments [39, 41]. This discrepancy might be related strictly to the type of injury and the therapeutic strategy used in our work. As the BDNF-rich Lin^−^BMCs are administered intravitreally and because they migrate to the RPE/photoreceptor junction, the released BDNF might consequently induce the TrkB expression selectively at a location close to the specific ligand.

In conclusion, we have confirmed previous studies showing that acutely injured retinas can incorporate Lin^−^BMCs from the vitreous chamber. Our data indicate that BM-derived cells might play a protective role in retinal degeneration by stimulating retinal cell survival. We propose a working model in which transplanted Lin^−^BMCs constitutively secrete BDNF that modulate the retinal microenvironment toward an antiapoptotic balance and in which they clean up the accumulated debris in the subretinal space. We suggest that the transplantation of Lin^−^BMCs might be a possible future route for protection of the retina and limiting retinal injury. The current display of protective effects after BMC transplantation opens up new horizons in the field of ophthalmology and may provide a simple but effective new strategy for clinical therapy in patients with retinal degenerative disorders.

## Supplementary Material

The supplementary materials contain supplementary tables representing the results obtained from RNA arrays performed for global gene expression analysis in retinas collected at day 7 after Lin-BMCs transplantation into murine eyes with induced acute chemical retinal injury.
Table S1: Top 25 downregulated genes in retinas injected with Lin-BMCs compared to retinas from PBS-treated eyes on the 7th day post NaIO_3_-injury.
Table S2: Top 25 upregulated genes in retinas injected with Lin-BMCs compared to retinas from PBS-treated eyes on the 7th day post NaIO_3_-injury.
