# Supplementary material for: Neuroprotective and Antiapoptotic Activity of Lineage-Negative Bone Marrow Cells after Intravitreal Injection in a Mouse Model of Acute Retinal Injury
Source: Stem Cells Int. 2015 Feb 24;2015:620364. doi: 10.1155/2015/620364 (PMC4354968; doi:10.1155/2015/620364)
Supplement: Supplementary file 1 — The supplementary materials contain supplementary tables representing the results obtained from RNA arrays performed for global gene expression analysis in retinas collected at day 7 after Lin-BMCs transplantation into murine eyes with induced acute chemical retinal injury. Table S1: Top 25 downregulated genes in retinas injected with Lin-BMCs compared to retinas from PBS-treated eyes on the 7th day post NaIO3-injury. Table S2: Top 25 upregulated genes in retinas injected with Lin-BMCs compared to retinas from PBS-treated eyes on the 7th day post NaIO3-injury. [file 620364.f1.pdf]

Table S1: Top 25 down-regulated genes in retinas injected with Lin^{-}BMCs compared to retinas from PBS-treated eyes on the 7th day post $NaIO_3$-injury.

Probe ID	Gene Symbol	log_2(right eye/left eye)	Gene Name	Entrez GeneID
10342383	Rab14	−5,05	RAB14, member RAS oncogene family	68365
10340656	Derl1	−4,97	Der1-like domain family, member 1	67819
10341530	Ddb1	−4,43	damage specific DNA binding protein 1	13194
10569017	Ifitm3	−4,41	interferon induced transmembrane protein 3	66141
10341637	Psap	−4,41	prosaposin	19156
10338802	Psmb5	−4,33	proteasome (prosome, macropain) subunit, beta type 5	19173
10343152	Anapc1	−4,32	anaphase promoting complex subunit 1	17222
10338683	Arfgef1	−4,28	ADP-ribosylation factor guanine nucleotide-exchange factor 1 (brefeldin A-inhibited)	211673
10338120	Psma1	−4,24	proteasome (prosome, macropain) subunit, alpha type 1	26440
10340293	Rab7	−4,09	RAB7, member RAS oncogene family	19349
10342263	Rragc	−4,09	Ras-related GTP binding C	54170
10344078	Itch	−4,04	itchy, E3 ubiquitin protein ligase	16396
10480734	Ptgds	−3,98	prostaglandin D2 synthase (brain)	19215
10475414	B2m	−3,93	beta-2 microglobulin	12010
10511363	Penk	−3,78	preproenkephalin	18619
10523717	Spp1	−3,76	secreted phosphoprotein 1	20750
10365482	Timp3	−3,75	tissue inhibitor of metalloproteinase 3	21859
10362896	Cd24a	−3,43	CD24a antigen	12484
10481627	Lcn2	−3,17	lipocalin 2	16819
10485466	Cat	−2,76	catalase	12359
10441815	Sod2	−2,34	superoxide dismutase 2, mitochondrial	20656
10449303	Bak1	−2,18	BCL2-antagonist/killer 1	12018
10467139	Lipa	−2,76	lysosomal acid lipase A	16889
10536494	Cav2	−2,69	caveolin 2	12390
10488415	Cst3	−2,69	cystatin C	13010

Table S2: Top 25 up-regulated genes in retinas injected with Lin$^-$BMCs compared to retinas from PBS-treated eyes on the 7th day post $NaIO_3$-injury.

Probe ID	Gene Symbol	$\log_2$(right eye/left eye)	GeneName	Entrez GeneID
10377429	Tmem107	5,23	transmembrane protein 107	66910
10598087	mt-Nd6	4,85	mitochondrially encoded NADH dehydrogenase 6	17722
10598071	mt-Cytb	4,39	mitochondrially encoded cytochrome b	17711
10565811	Rps3	2,93	ribosomal protein S3	27050
10560746	Nlrp4e	2,74	NLR family, pyrin domain containing 4E	446099
10566684	Olfr488	2,6	olfactory receptor 488	258727
10566387	Dub3	2,56	deubiquitinating enzyme 3	625530
10447647	Gpr31c	2,52	G protein-coupled receptor 31, D17Leh66c region	436440
10388286	Olfr395	2,5	olfactory receptor 395	259007
10383632	Sfi1	2,47	Sfi1 homolog, spindle assembly associated (yeast)	78887
10566692	Olfr502	2,46	olfactory receptor 502	258734
10389668	Olfr462	2,38	olfactory receptor 462	258406
10554731	Olfr293	2,32	olfactory receptor 293	257906
10340689	Zfp518a	2,32	zinc finger protein 518A	72672
10547171	Olfr215	2,3	olfactory receptor 215	258438
10493860	Sprr2b	2,27	small proline-rich protein 2B	20756
10556131	Olfr483	2,27	olfactory receptor 483	258730
10566611	Olfr707	2,23	olfactory receptor 707	194433
10556129	Olfr481	2,21	olfactory receptor 481	258927
10556154	Olfr506	*2,2*	olfactory receptor 506	258215
10515828	Olfr1335	2,2	olfactory receptor 1335	435804
10471782	Olfr368	2,16	olfactory receptor 368	258371
10367507	Olfr792	2,16	olfactory receptor 792	258150
10566626	Olfr715	2,15	olfactory receptor 715	258776
10471745	Olfr342	2,11	olfactory receptor 342	258950
10544436	Olfr458	2,1	olfactory receptor 458	258436
